# Psychometric Evaluation of the Krogh-Poulsen Test for the Diagnosis of the Temporomandibular Disorders

**DOI:** 10.3390/diagnostics11101876

**Published:** 2021-10-12

**Authors:** Alfonso Javier Ibáñez-Vera, Roger Alonso-Royo, Carmen María Sánchez-Torrelo, Noelia Zagalaz-Anula, Jesús López-Collantes, Rafael Lomas-Vega

**Affiliations:** 1 Department of Health Sciences, Campus de las Lagunillas, University of Jaén, 23071 Jaén, Spain; ajibanez@ujaen.es (A.J.I.-V.); rlomas@ujaen.es (R.L.-V.); 2 FisioMedic Clinic, Dos Hermanas, 41701 Sevilla, Spain; rar00032@red.ujaen.es (R.A.-R.); fisiomedic.dh@gmail.com (C.M.S.-T.); 3 Dental Medical Center Drs. López Collantes, Dos Hermanas, 41701 Sevilla, Spain; citas@lopezcollantes.es

**Keywords:** craniomandibular disorders, temporomandibular joint disorders, principal component analysis, reproducibility of results, data correlation, ROC curve

## Abstract

The Krogh-Poulsen Test is a classic instrument to measure dysfunction of the stomatognathic system whose psychometric properties are unknown. This study aimed to evaluate the psychometric properties of the Krogh-Poulsen Test for the diagnosis of temporomandibular disorders (TMDs). A cross-sectional study was designed, including 119 patients (63 patients with TMD and 56 healthy controls). Factorial validity, inter-rater reliability, error of measurement, diagnostic validity of the Krogh-Poulsen Test, and concurrent validity were analyzed. The Krogh-Poulsen Test showed a three-factor structure. The inter-rater agreement could be considered very good with a kappa index of 0.87 (95% CI 0.83–0.90) and Standard Error of Measurement of 0.79. Correlations were strong with other orofacial instruments, moderate with instruments measuring TMD-related disorders such as neck pain, headache, or dizziness, and poor with generic quality of life instruments. The Area Under the Curve ROC was 0.928 showing, for a cut-off point >1, a sensitivity of 90.48 (95% CI 80.4–96.4) and a specificity of 85.71 (95% CI 73.8–93.6) for the diagnosis of TMD disorders. The Krogh-Poulsen Test showed a three-factor structure, very good inter-rater reliability, a strong correlation with other orofacial instruments, and an excellent capacity to discriminate between patients with or without TMD.

## 1. Introduction

Temporomandibular disorder (TMD) is a dysfunction that affects the temporomandibular joint (TMJ), masticatory muscles, and associated structures [1]. Signs and symptoms of TMD may include impaired jaw function, malocclusion, deviation from the midline on opening or closing the jaw, joint noises, limited range of motion, locking, and pain [2]. The International Association for the Study of Pain (IASP) defines orofacial pain as a frequent form of pain perceived in the face and/or oral cavity that may be caused by diseases or disorders of regional structures, dysfunction of the nervous system, or through referral from distant sources [3].TMD has a multifactorial etiology that is influenced by the initiation and/or perpetuation of cofactors such as bruxism [4,5]. The global prevalence of temporomandibular joint and muscle disorder is between 5 and 12% [6], affecting women more than twice as often as men [7].

The International Network for Orofacial Pain and Related Disorders Methodology recommends the use of the Diagnostic Criteria for Temporomandibular Disorders (DC/TMD) [8] for TMD diagnosis, which evaluates muscle and joint pain, measurements of the different movements of the TMJ, type of bite, opening pattern, headaches in the last 30 days, noises (clicks and crackles), joint blockages, pain on palpation, and TMJ and muscle pathologies. The DC/TMD protocol is the gold standard for TMD diagnosis; however, it is a complex test that requires training for its correct application and takes a long time to administer.

In 1969, Krogh-Poulsen developed a clinical examination to establish the categories that precede the diagnosis of dysfunction, which is relevant for prevention. The categories are healthy patient, at risk, disturbed, and sick [9]. This test consists of nine items and assesses the masticatory muscles, TMJ, and dental occlusion [10]. In 1999, Cornejo-Salazar evaluated the sensitivity and specificity of the Krogh-Poulsen Test, using the Helkimo Index [11,12] as the gold standard, for TMD diagnostic procedures. The results indicated a sensitivity of 78% and a specificity of 100%, providing evidence for the diagnostic value of the Krogh-Poulsen Test and of its greater utility in the diagnosis of truly healthy individuals [13].

The Krogh-Poulsen Test is internationally recognized and used to measure dysfunction of the stomatognathic system. However, to date, it has not been validated. For this reason, this study aimed to assess and test the psychometric properties of the Krogh-Poulsen Test in patients with TMD.

## 2. Materials and Methods

### 2.1. Study Design

A cross-sectional observational study was conducted to validate the Krogh-Poulsen Test. The study complied with the ethical principles for biomedical research in humans presented in the Declaration of Helsinki and authorized by the Ethics of the University of Jaen (code ABR.20/3TFM, date of approval 27 April 2020). All participants signed an informed consent at enrollment.

### 2.2. Participants

The participants were recruited at the FisioMedic Clinic and the Drs. Lopez Collantes Dental Medical Center, both in the city of Dos Hermanas (Seville, Spain). The subjects were contacted by telephone and interviewed at the FisioMedic Clinic, where they were informed about their rights and the interventions to be performed. Their acceptance to participate in the study was formalized by signing an informed consent.

The selection criteria included participants over 18 years old who were diagnosed with TMD with the DC/TMD test at the time of assessment. Those participants who did not have the necessary capacities to carry out the assessments required for the study were excluded (participants unable to read or unable to hear the instructions). In addition, a control group of equivalent participants unaffected by TMD according to the DC/TMD was selected to analyze the accuracy of the Krogh-Poulsen Test for the diagnosis of TMDs.

### 2.3. Sample Size

Regarding the sample size calculation, the general recommendation for the calculation of the sample of questionnaire validation studies was followed, with a minimum of 5 subjects and an optimal number of 10 subjects per questionnaire item [14]. As the Krogh-Poulsen Test comprises 9 items, a sample of 63 affected subjects was used. Finally, in order to discriminate the subjects affected by TMD from healthy subjects, a control group of 56 participants unaffected by TMD was also included, comprising a total sample of 119 participants.

### 2.4. Measurements

The sociodemographic data collected included age in years, height in meters, weight in kilograms (both measured with Detecto^®^, model 2391, Webb City, MO, USA), Body Mass Index (BMI) calculated from weight and height, gender, educational level, alcoholic use [15], and smoking habit [16]. Finally, we measured physical activity, considering whether the participant performed at least 150 min of physical activity per week or not [17].

The diagnostic validity of Krogh-Poulsen was assessed with respect to the gold standard DC/TMD, which is a tool that requires specific training and materials for its application. It consists of 12 items that assess muscle and joint pain, pain in the TMJ movement, headache, bite issues, noises, and jaw blocks. Once this information is collected, a diagnostic tree is used to obtain a diagnosis. This assessment has an inter-examination reliability of 0.85, a specificity of 0.98, and sensitivity of 0.86 [8].

The Krogh-Poulsen Test was the diagnostic tool under study. This is a hetero-administered assessment for TMD that comprises nine items that can be answered as “Yes” or “No” (Supplementary Table S1). The subject was considered healthy from the responses obtained if all the responses were negative, affected if it had a positive response, at risk of TMD if the subject had two positive responses, and dysfunctional if the subject had three or more positive responses [9].

For concurrent validity analysis, other validated tests were used, such as the Helkimo Index, a tool composed of five items with different responses that score 0, 1, or 5. The sum of the items is categorized as unaffected (0 points), slight (1–9 points), moderate (10–19 points), and severe (20–25 points) [12,18]. The Fonseca Anamnestic Index (FAI) and its short version (SFAI) were also used. The FAI is a test that consists of 10 items [19,20] (5 in the short version) [21] that can be answered as “Yes,” “No,” and “Sometimes,” scoring 10, 0, and 5, respectively. The sum of the scores of the FAI is categorized as unaffected (0–15 points), mild (20–40 points), moderate (45–65 points), and severe (70–100) [19], while the SFAI categorizes only unaffected (0–15 points) and affected (20–50 points) subjects [21].

To assess orofacial and neck self-perceived pain, the Numeric Pain Rating Scale (NPRS) was used. This is a pain intensity scale self-implemented that scores from 0 (absence of pain) to 10 points (maximum pain) [22]. The Neck Disability Index (NDI) was also used, which assesses disability due to neck pain. This instrument is made up of 10 items that can be answered by 6 different responses that score 0, absence of disability, and 5, maximum disability [23].

Owing to the relationship among neck pain, TMD, headache, and dizziness, both the Headache Impact Test (HIT-6) and the Dizziness Handicap Inventory (DHI) were used. The HIT-6 was used for the evaluation of impact of headache in these participants and consists of a self-implemented test composed of six items with different answer options that score 6, 8, 10, and 13 points [24]. The DHI assessed the disability due to vertigo and balance problems. It is made up of 25 items that can be answered as “Yes,” “No,” and “Sometimes,” scoring 4, 0, and 2, respectively. The questionnaire evaluates vertigo according to different origins, considering a Physical Component that includes items from 1 to 9, an Emotional Component from 10 to 16 and a Functional Component from 17 to 25 [25,26].

Finally, the Short Form Health Survey Questionnaire (SF-12) was used to assess the general quality of life, because of its consideration as a reference instrument. This questionnaire consists of 12 items that are divided into a Physical Component and Mental Component [27].

### 2.5. Procedure

The subjects were contacted by telephone for a meeting at the FisioMedic Clinic, where the research was carried out. There, they were informed of the conditions of the study. If they accepted participation, this was formalized by signing an informed consent document. Once accepted into the study, the subjects filled in the questionnaires and were evaluated with the Krogh-Poulsen Test by two experienced professionals well-trained in the use of the questionnaires and tests previously described.

### 2.6. Data Analysis

Data were described by means and standard deviation for continuous variables and by frequencies and percentages for categorical variables. Normality of the data distribution was tested with the Kolmogorov-Smirnov Test. We worked with a confidence level of 95%.

For the factorial analysis we used FACTOR (Unrestricted Factor Analysis Release Version 10.4.01, April 2016, Rovira i Virgili University Tarragona, Tarragona, Spain). Because of the dichotomy of the items, we used the matrix of polychoric correlations. Principal components analysis was selected for the extraction of factors, and varimax for factor rotation. Measurement of unidimensionality was made using the methodology of Ferrando and Lorenzo-Seva [28]. The criteria for unidimensionality were unidimensional congruences (UniCo) larger than 0.95, an explained common variance (ECV) larger than 0.85 and mean of item residual absolute loadings (MIREALs) lower than 0.300. Adequacy of correlation matrix was measured by calculation of the matrix determinant, Bartlett test, and Kaiser-Meyer-Olkin (KMO) test.

For the analysis of reliability, concurrent validity and discriminant validity, MedCalc^®^ Statistical Software version 19.8 (MedCalc Software Ltd., Ostend, Belgium; https://www.medcalc.org; 2021 was used. Weighted kappa was used for the analysis of inter-rater agreement. Weighted kappa is asymptotically equivalent to an intraclass correlation coefficient [29]. Following the recommendations of Landis and Koch [30], agreement was considered null when kappa = 0.21–0.40, moderate if kappa = 0.41–0.60, substantial if kappa = 0.61–0.80 and very good if kappa = 0.81–1.00. The standard error of measurement (SEM) was calculated as the baseline standard deviation (SD) (σbase) minus the square root of (1-Rxx), where Rxx is the reliability index (kappa). The minimum detectable change (MDC) was calculated from the SEM formula as follows: MDC95 = 1.96 × σbase × $\sqrt{\left(1\;-\;ICC\right)}$, where 1.96 is the z-value corresponding to the 95% confidence interval (MDC95). The MDC provides a good tool for translating the reliability index into units of change in the instrument. Bland-Altman plots were obtained for the calculation of the limits of agreement. Correlation coefficients of Spearman’s rho between the Krogh-Poulsen Test total score and the other instruments were calculated to evaluate concurrent validity. Following Cohen’s criteria [31], correlations lower than 0.30 were considered poor, moderate between 0.30–0.50, and strong with correlations of more than 0.50.

The capacity of the Krogh-Poulsen Test to discriminate between TMD patients and healthy subjects was calculated using receiver operating characteristic (ROC) curves. The subject classification as TMD patients or healthy controls was carried out based on the diagnostic criteria of the DC/TMD protocol, and the total score obtained in the Krogh-Poulsen Test was evaluated as a variable. In the ROC curve, the fraction of true positives (sensitivity) was represented as a function of the fraction of false positives for different cut-off points. The area under the curve (AUC) was also calculated as a measure of the ability of the score to discriminate between the two diagnostic groups (TMD patients or healthy subjects). The AUC was considered statistically significant when the 95% confidence interval did not include 0.5 [32]. Values between 0.5 and 0.7 indicated low accuracy, values between 0.7 and 0.9 indicated good accuracy, and values greater than 0.9 indicated high accuracy [33].

## 3. Results

Data of the sample and the groups of participants are shown in Table 1. The participants in the TMD group were most commonly female with university studies. There were no significant differences for the other variables.

### 3.1. Factorial Analysis

Indicators of unidimensionality were UniCo = 0.873 (95% CI 0.764–0.976), ECV = 0.729 (95% CI 0.613–0.826), and MIREAL = 0.355 (95% CI 0.315–0.442), so the scale could not be considered as one-dimensional. We tested the best dimensionality of the scale, and the structure based on three factors showed an adequacy of the tetrachoric correlation matrix with a determinant of the matrix = 0.000005836548800. Bartlett’s statistic was statistically significant (*p* = 0.000010), and the KMO test was 0.887 (95% CI 0.871–0.924), which could be considered good. The explained variance based on eigenvalues was 75%. The generally acceptable structure showed two factors composed of items 1–6 and items 7–9. Over the optimal three-factor structure, item 2 formed the first factor (Table 2).

### 3.2. Reliability and Inter-Rater Agreement

**Figure 1:**
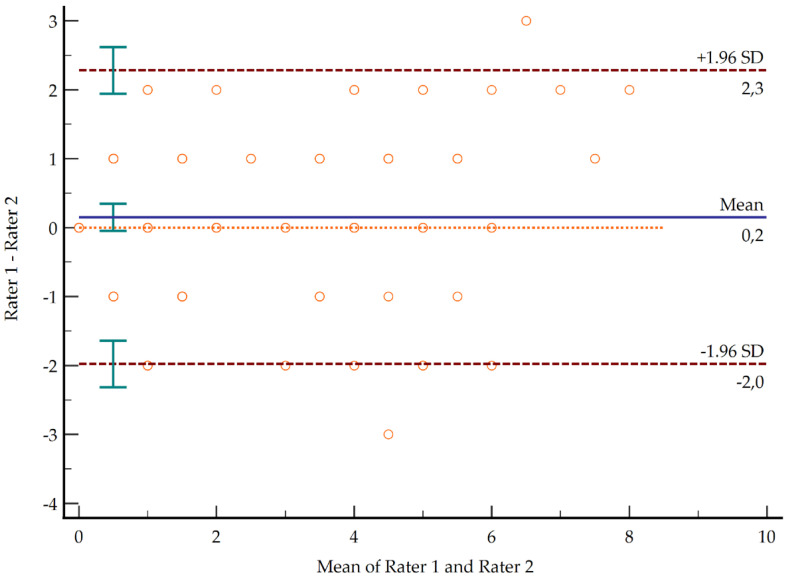
Bland-Altman plot showing the limits of agreement with a 95% confidence level.

The total score showed a kappa index of 0.87 (95% CI 0.83–0.90), which could be considered a very good agreement. Based on this index, SEM was 0.79, and MDC was 1.55. These findings mean that in a total score varying by 0–9 points, the error can be less than 1 point and the minimal change detected can be less than 2 points. The Bland-Altman analysis showed that the limits of agreement varied between ±2 points in 95% of the cases (Figure 1).

### 3.3. Concurrent Validity

The correlation between the total score of the Krogh-Poulsen Test and other health measurements (Table 3) was considered strong when compared with other orofacial pain measures, moderate when compared with instruments measuring TMD-related disorders such as headache, neck pain, or dizziness, and the relationship with general measures of health status or quality of life was poor.

### 3.4. Discriminant Validity

**Figure 2:**
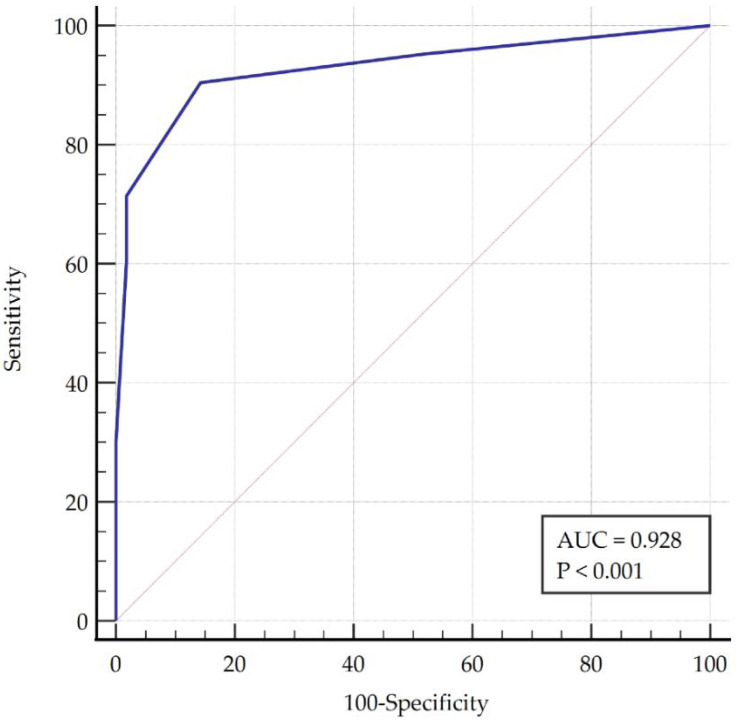
Receiver operating characteristic (ROC) curve showing an excellent capacity to discriminate between patients with or without temporomandibular disorders. AUC: area under the curve.

The ROC curve analysis showed an AUC of 0.928 that was considered high accuracy (Figure 2). A cut-of-point >1 in the Krogh-Poulsen Test score showed a sensitivity of 90.48 and a specificity of 85.71 for the diagnosis of TMD disorders (Table 4).

## 4. Discussion

The present study analyzed the clinometric properties of the Krogh-Poulsen Test, which was designed as a quick diagnostic tool for TMD. The Krogh-Poulsen Test allows a complete assessment of the stomatognathic system and takes considerably less time with respect to the DC/TMD. According to our study, the Krogh-Poulsen Test is an excellent instrument for discriminating affected from unaffected patients. Our study used the Krogh-Poulsen Test on 119 participants divided into 2 comparable groups: one group with 63 affected by TMD and another group with 56 healthy controls. The only difference between groups was the higher proportion of females, a common observation in TMD studies [12,18,34].

Created by Dr. Krogh-Poulsen, this instrument was a well-known and commonly used diagnostic index in the 1970s and 1980s [9,35,36,37], discontinued with time owing to the lack of colorimetric analysis. In 1978, Kerschbaum et al. pointed out that the Krogh-Poulsen Test could detect functional disturbances in only 12% of 361 patients with removable partial protheses, suggesting poor reliability, objectivity, and validity of the index [38]. This contrasted completely with our findings, which revealed good psychometric properties for the diagnosis of TMDs. Unfortunately, the antiquity of the article did not allow us to assess the methods used, and therefore we could not identify the reasons for this discrepancy. However, it may be due to the different study populations, as those in our study were non-users of prostheses.

In 1999, a study analyzed the sensibility and specificity of the Krogh-Poulsen Test in 80 participants between 15 and 50 years old using the Helkimo Index as the gold standard [13]. The data of that study showed a sensibility of 0.78 and a specificity of 100% in TMD diagnosis, compared with the Helkimo Index. These results agreed with the excellent ones obtained in the present study about the reliability and MDC. Based on this data, the index can be considered very useful for TMD diagnostics. In our study, the gold standard was DC/TMD, which is the currently accepted instrument for TMD diagnosis. Additionally, we analyzed the concurrent validity of the Krogh-Poulsen Test with the Helkimo Index, obtaining a strong correlation.

Individual analysis of some of the items of the Krogh-Poulsen Test has been performed. Konan et al. (2003) compared the bite test item, which differentiates whether the pain is due to muscles or to the TMJ, with computed tomography images, detecting a high degree of correlation between a positive result for joint pain and radiological abnormalities of the joint. Regarding the positive results for muscle pain, 70% of these patients did not present any radiological abnormality of the joint. That study provided evidence for the high validity of the Krogh-Poulsen Test in relation to radiologic studies, strengthening our findings.

The clinical importance of our findings relies on the validation of a classic diagnostic index that is still often used in dentistry and physiotherapy and saves time when diagnosing, compared with the gold standard test. The Krogh-Poulsen Test has a high correlation with other diagnostic instruments for TMD and a moderate correlation with instruments that measure related disorders. This will allow primary care physicians and other professionals to perform a quick and high-quality assessment, enhancing adequate treatment by referring to the appropriate health professional. Despite this, the study has some limitations: firstly, the sample was recruited in a single location and presented a higher proportion of female participants, although this proportion is common in TMD studies. Thus, we encourage further multicenter studies to increase the generalizability of our results. In addition, although the prevalence of the disease does not affect the sensitivity and specificity values, it does affect other predictive values [39]. Thus, since the proportion of patients in our sample was higher than the prevalence of the disease in the general population, positive predictive value and negative predictive value must be taken with caution since the positive predictive value may possibly be overvalued in our study. Further studies should also investigate the ability to discriminate between different TMD conditions.

## 5. Conclusions

The Krogh-Poulsen Test is a valid instrument for measuring temporomandibular disorders and enables the differentiation between affected and healthy patients, presenting a high correlation with other TMD diagnostic instruments and moderate correlation with instruments that measure associate disorders such as headache, dizziness, vertigo, and neck function. Inter-rater reliability of the Krogh-Poulsen Test can be considered very good.

## Figures and Tables

**Table 1:** Sociodemographic characteristics of the sample and groups.

		TMD	*n* = 63	Healthy	*n* = 56	All	*n* = 119
Gender	Female	59	93.7%	32	57.14%	91	76.5%
	Male	4	6.3%	24	42.86%	28	23.5%
Job	Active	12	19.0%	14	25.00%	26	21.8%
	Not active	51	81.0%	42	75.00%	93	78.2%
Study Level	Primary	7	11.1%	14	25.00%	21	17.6%
	Secondary	28	44.4%	30	53.57%	58	48.7%
	University	28	44.4%	12	21.43%	40	33.6%
Physical Activity	No	27	42.9%	20	35.71%	47	39.5%
	Yes	36	57.1%	36	64.29%	72	60.5%
Income	<20,000	35	55.6%	36	64.29%	71	59.7%
	>20,000	28	44.4%	20	35.71%	48	40.3%
Smoker	Non-smoker	43	68.3%	34	60.71%	77	64.7%
	Smoker	8	12.7%	7	12.50%	15	12.6%
	Occasional smoker	6	9.5%	7	12.50%	13	10.9%
	Ex-smoker	6	9.5%	8	14.29%	14	11.8%
Drinker	Non-drinker	21	33.3%	21	37.50%	42	35.3%
	Regular drinker	3	4.8%	3	5.36%	6	5.0%
	Occasional drinker	39	61.9%	32	57.14%	71	59.7%
Age (years)		43.19	(12.62)	47.64	(14.87)	45	(13.86)
Weight (kilograms)		68.90	(14.07)	77.86	(19.22)	72.83	(17.05)
Height (meters)		1.61	(0.07)	1.65	(0.09)	1.63	(0.09)
Body Mass Index		26.69	(6.72)	28.48	(7.10)	27	(6.91)

TMD: Temporomandibular Disorders.

**Table 2:** Rotated matrix component showing factorial loads of each item on each factor.

Items	Component		
	1 ^a^	2 ^a^	3 ^a^
I. Mouth opening under 40 mm	0.149	−0.072	0.697
II. Deviation in mandibular movement during opening or closing	0.942	0.058	0.112
III. Discomfort at masticatory muscle palpation	0.087	0.238	0.859
IV. Pain at pressing temporomandibular joint	0.068	0.286	0.831
V. Clicks or crackles during joint movement	0.456	−0.002	0.698
VI. Obstacles or blockages during joint movement	0.161	0.319	0.682
VII. Centric relation and intercuspation	0.471	0.657	0.312
VIII. Anterior displacement over 1 mm at retrusion from maximum intercuspation	−0.184	0.887	0.028
IX. Lateral displacement over 1 mm at retrusion	0.217	0.885	0.245

^a^ Factors obtained from Krogh-Poulsen Test factor analysis.

**Table 3:** Concurrent validity of the Krogh-Poulsen Test and different instruments measuring pain and health status.

	Rho Coefficient	*p*-Value	Correlation
Fonseca Anamnestic Index	0.702	<0.001	Strong
Fonseca Anamnestic Index, Short	0.675	<0.001	Strong
Helkimo Clinical Dysfunction Index	0.710	<0.001	Strong
Numerical Rating Scale, Orofacial Pain	0.865	<0.001	Strong
Headache Impact Test, 6 items	0.329	<0.001	Moderate
Dizziness Handicap Inventory, Functional	0.322	<0.001	Moderate
Dizziness Handicap Inventory, Emotional	0.292	0.001	Poor
Dizziness Handicap Inventory, Physical	0.389	<0.001	Moderate
Dizziness Handicap Inventory, Total Score	0.377	<0.001	Moderate
Neck Disability Index	0.423	<0.001	Moderate
Numerical Rating Scale, Neck Pain	0.399	<0.001	Moderate
Physical Component Summary	−0.076	0.414	Poor
Mental Component Summary	−0.197	0.032	Poor

**Table 4:** Predictive values of Krogh-Poulsen Test for temporomandibular disorders.

Criterion	Sensitivity	95% CI	Specificity	95% CI	+LR	95% CI	−LR	95% CI	+PV	95% CI	−PV	95% CI
>1	90.48	80.4–96.4	85.71	73.8–93.6	6.33	3.3–12.1	0.11	0.05–0.2	87.7	78.9–93.2	88.9	78.8–94.5

95% CI: 95% confidence interval; +LR: positive likelihood ratio; −LR: negative likelihood ratio; +PV: positive predictive value; −PV: negative predictive value.

## Data Availability

Data is available under request to the corresponding author.

## Supplementary Materials

Supplementary material is available online at https://www.mdpi.com/article/10.3390/diagnostics11101876/s1, Table S1: Krogh Poulsen Test.
