# Supplementary material for: Psychometric Evaluation of the Krogh-Poulsen Test for the Diagnosis of the Temporomandibular Disorders
Source: Diagnostics (Basel). 2021 Oct 12;11(10):1876. doi: 10.3390/diagnostics11101876 (PMC8534852; doi:10.3390/diagnostics11101876)
Supplement: Supplementary file 1 [file diagnostics-11-01876-s001.zip › diagnostics-1303581-supplementary.pdf]

Supplementary Material

Psychometric Evaluation of the Krogh-Poulsen Test for the Diagnosis of the Temporomandibular Disorders

Table S1. Krogh Poulsen Test.

Signs and Symptoms	Yes	No
I. Mouth opening under 40 mm		
II. Deviation in mandibular movement during opening or closing		
III. Discomfort at masticatory muscles palpation		
IV. Pain at pressing temporomandibular joint		
V. Clicks or crackles during joint movement		
VI. Obstacles or blockages while joint movement		
VII. Centric relation and intercuspation		
VIII. Anterior displacement over 1 mm at retrusion from maximum intercuspation		
IX. Lateral displacement over 1 mm at retrusion		

Unaffected ____________ No positive aspect
Disturbance ____________ One positive aspect
Risk ____________ Two positive aspects
Dysfunction ____________ Three positive aspects
Items 6 or 9 positive (them are decisive)
